# In vivo genome-wide analysis of multiple tissues identifies gene regulatory networks, novel functions and downstream regulatory genes for *Bapx1* and its co-regulation with *Sox9* in the mammalian vertebral column

**DOI:** 10.1186/1471-2164-15-1072

**Published:** 2014-12-05

**Authors:** Sumantra Chatterjee, V Sivakamasundari, Sook Peng Yap, Petra Kraus, Vibhor Kumar, Xing Xing, Siew Lan Lim, Joel Sng, Shyam Prabhakar, Thomas Lufkin

**Affiliations:** Stem Cell and Developmental Biology, 60 Biopolis Street, Singapore, 138672 Singapore; Computational and Mathematical Biology, Genome Institute of Singapore, 60 Biopolis Street, Singapore, 138672 Singapore; Department of Biology, Clarkson University, 8 Clarkson Avenue, Potsdam, NY 13699 USA

**Keywords:** Gene regulatory network, Expression profiling, Bapx1, Sox9, ChIP-Seq, FACS, Microarray, EGFP

## Abstract

**Background:**

Vertebrate organogenesis is a highly complex process involving sequential cascades of transcription factor activation or repression. Interestingly a single developmental control gene can occasionally be essential for the morphogenesis and differentiation of tissues and organs arising from vastly disparate embryological lineages.

**Results:**

Here we elucidated the role of the mammalian homeobox gene *Bapx1* during the embryogenesis of five distinct organs at E12.5 - vertebral column, spleen, gut, forelimb and hindlimb - using expression profiling of sorted wildtype and mutant cells combined with genome wide binding site analysis. Furthermore we analyzed the development of the vertebral column at the molecular level by combining transcriptional profiling and genome wide binding data for *Bapx1* with similarly generated data sets for *Sox9* to assemble a detailed gene regulatory network revealing genes previously not reported to be controlled by either of these two transcription factors.

**Conclusions:**

The gene regulatory network appears to control cell fate decisions and morphogenesis in the vertebral column along with the prevention of premature chondrocyte differentiation thus providing a detailed molecular view of vertebral column development.

**Electronic supplementary material:**

The online version of this article (doi:10.1186/1471-2164-15-1072) contains supplementary material, which is available to authorized users.

## Background

Vertebrate development is a complex, sequential process, which is guided and controlled by a large number of transcription factors acting in a spatio-temporal manner. Many of these transcription factors are pleiotropic in nature, expressing and functioning in multiple tissues simultaneously. Pleiotropy is a feature of some genes, which refers to the ability to affect multiple, distinct and unrelated phenotypes and has important implications in development, disease and evolution [1–4]. Although pleiotropy has been studied over a period of time for genes involved in various biological functions, very few in-depth molecular studies have been conducted looking at a single gene in detail covering its roles in multiple tissues and trying to understand the independent and /or overlapping molecular mechanisms involved in its disparate functions.

The mammalian homeobox gene *Bapx1* (*Nkx3.2*) encodes a transcription factor that belongs to the eponymous *Nkx*[5] gene family ortholog of the *Drosophila bap* gene [6, 7]. During mouse embryogenesis *Bapx1* is expressed in five distinct tissues - vertebral column, spleen, gut, forelimb and hindlimb, yet appears to have a unique function in only a subset of these. *Bapx1* null mice are affected by lethal skeletal dysplasia, with severe malformation or absence of specific bones of the vertebral column and cranial bones of mesodermal origin. The most severely affected parts of the skeleton are the ventral structures, which are in close proximity to the notochord. *Bapx1* null mutants also show visceral mesoderm defects leading to asplenia [8–12]. The *Bapx1* null embryos exhibit a shortened anterior gut segment and a loss of the pyloric constriction [11]. Although *Bapx1* is expressed in the developing limbs there is no overt limb phenotype in the loss-of-function mutants but a *Bapx1* gain-of-function mouse model exhibits preaxial polydactyly and hypoplasia of the tibia [13].

Since *Bapx1* is expressed in five distinct tissues it was imperative to segregate these tissues as well as the specific cell type within the tissues expressing *Bapx1* for a detailed understanding of its molecular mechanism. We achieved this by targeting the *Bapx1* locus with enhanced green fluorescent protein (EGFP) driven by an IRES (*Bapx1*^*+/tm2.Tlu*^) into the 3’UTR of *Bapx1* allowing for mice of a wildtype genotype expressing both Bapx1 and EGFP proteins or by inserting *EGFP* immediately after the translational start codon of *Bapx1* (*Bapx1*^*tm4.Tlu/tm4.Tlu*^) to create a null genotype. In both cases *EGFP* was expressed under the control of endogenous *Bapx1* transcriptional regulatory elements. This enabled us to isolate specifically only cells expressing *Bapx1* from these heterogeneous tissues using fluorescence activated cell sorting (FACS), and to compare the expression profiles of *Bapx1* target genes in wildtype versus loss-of-function embryos as a first step to understanding the pleiotropic roles of *Bapx1*.

It is essential when unraveling the role of a transcription factor in directing organogenesis to take the complementary and supporting roles played by other transcription factors that are active in the same cells into consideration. To understand such complementary roles we focused on the vertebral column, an organ unique to vertebrates providing body structure and support, which undergoes endochondral ossification of its cartilaginous structures to form bones. To decipher the possible molecular mechanism of chondrocyte differentiation and maturation in the vertebral column we co-analyzed the transcription factor *Sox9,* which has a major role in development and morphogenesis of chondrocytes [14, 15].

*Sox9* is a known master regulator in the osteo-chondrogenic lineage but its co-regulatory role with *Bapx1* has not been fully explored, though some evidence suggests that it lies in a regulatory cascade upstream of *Bapx1*[16]. To explore this, we performed similar expression profiling and genome wide binding studies in the vertebral column of E12.5 wildtype and *Sox9* null mice, to uncover its role independently, as well as decipher antagonistic and complementary co-regulatory roles between *Sox9* and *Bapx1*, by comparing unique and shared target genes. We uncovered 137 genes, which are regulated by both *Bapx1* and *Sox9* encompassing very different functions. Finally by combining the genome wide binding and transcriptional profiling data we have generated a gene regulatory network directed by *Bapx1* and *Sox9* in the mouse vertebral column at E12.5*.*

## Results

### Generation of transgenic mouse lines

**Figure 1:**
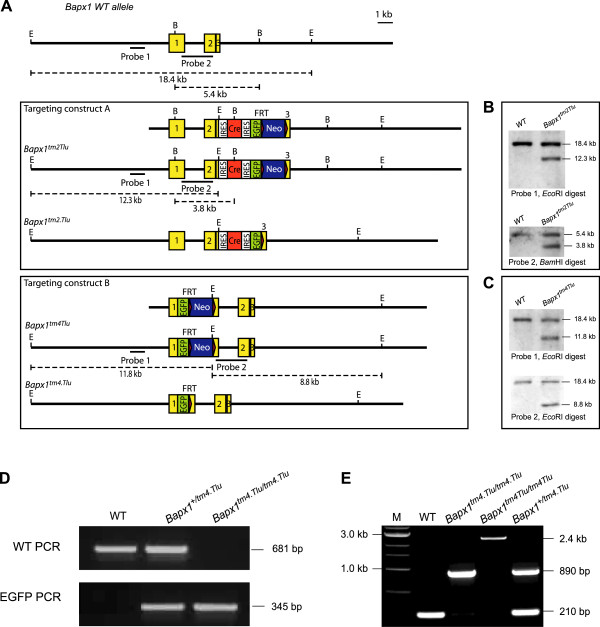
**Targeting constructs for** ***Bapx1*** **. (A)** Wildtype *Bapx1* allele and targeted *Bapx1* alleles producing EGFP via IRES in the wildtype condition (*Bapx1* ^tm2.Tlu^) and via the endogenous *Bapx1* promoter in the loss-of function condition (*Bapx1* ^tm4.Tlu^) as adapted from [17]. Southern blot with internal and external probes to verify the targeting event of the *Bapx1* ^tm2.Tlu^ **(B)** and *Bapx1* ^tm4.Tlu^ **(C)** alleles. Routine PCR genotyping to verify the wildtype and targeted allele **(D)** and the presence and absence of the neomycin cassette **(E)**.

**Figure 2:**
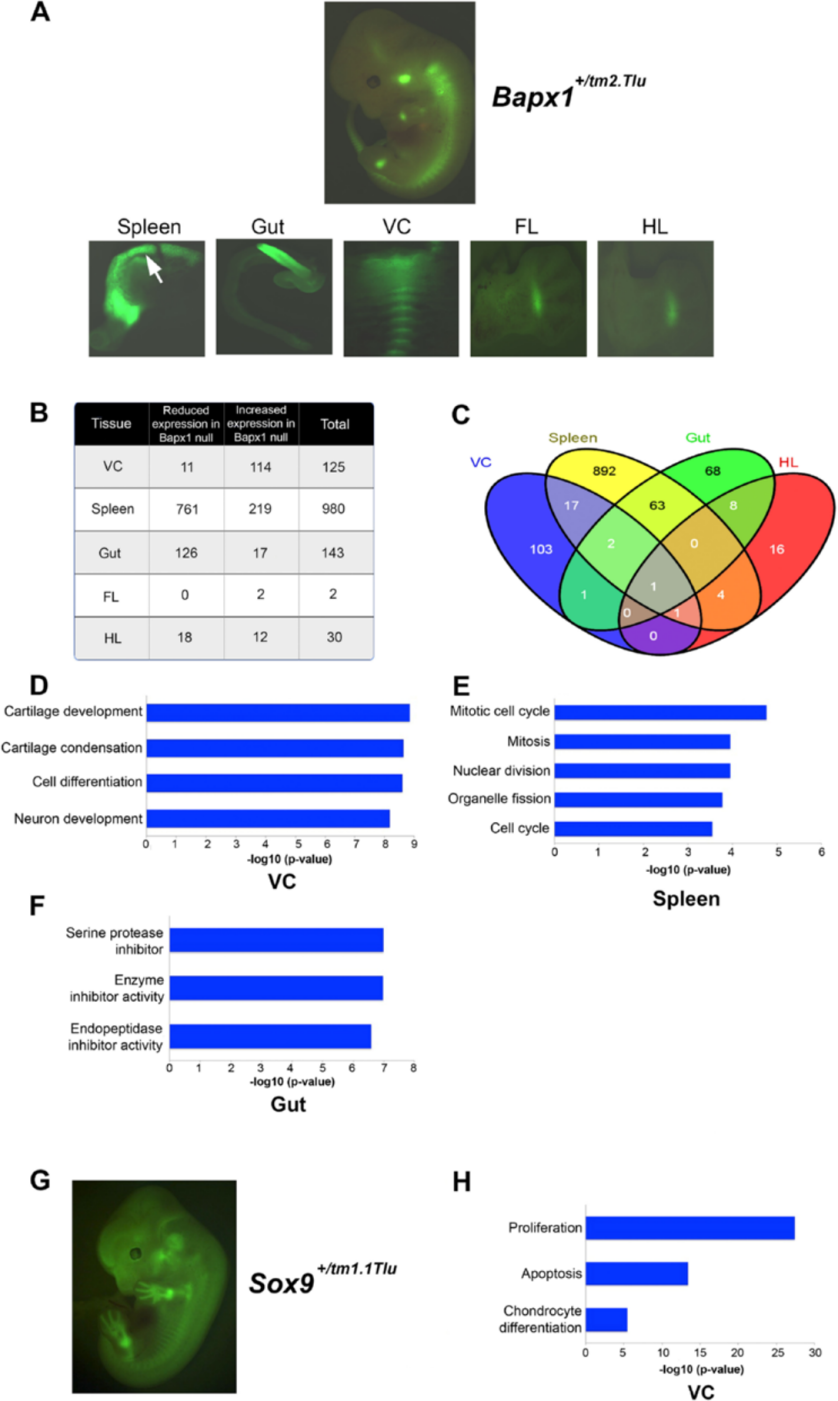
**Expression profiling of E12.5** ***Bapx1*** **and** ***Sox9*** **producing cells from embryos. (A)** Endogenous EGFP tagged E12.5 day whole embryo showing EGFP in domains of expression of *Bapx1* and the five distinct organs of expression- spleen, gut, vertebral column (VC), forelimb (FL) and hindlimb (HL). **(B)** Gene expression profiling of cells sorted from five organs in the wildtype embryo as compared to null showing Bapx1 can function as either an activator or repressor in these organs. **(C)** Venn diagram showing almost non-overlapping regulation in Bapx1 expressing organs. GO annotations of genes regulated by Bapx1 in vertebral column **(D)**, gut **(E)** and spleen **(F)** where the x-axis is -log10 (p-value). **(G)** E12.5 day whole embryo showing EGFP expression under the endogenous Sox9 promoter in expected domains. **(H)** GO annotations of genes regulated by *Sox9* in the vertebral column (VC) where the x-axis is -log10 (p-value).

With the aim of isolating *Bapx1* expressing cells by FACS for *in vivo* comparative gene expression analysis, we targeted the endogenous *Bapx1* locus with the enhanced green fluorescent protein (*EGFP*) reporter gene via homologous recombination in mouse embryonic stem (ES) cells, generating a *Bapx1* wildtype (*Bapx1*^*tm2.Tlu*^) and a *Bapx1* null (*Bapx1*^*tm4.Tlu*^) transgenic allele, both enabling expression of *EGFP* under control of the endogenous *Bapx1* promoter (Figure 1A adapted from [17]). *Bapx1*^*tm2.Tlu*^ was designed with the reporter linked via the internal ribosomal entry site (IRES) at the 3’ UTR of the *Bapx1* locus, forming a bicistronic system with two open reading frames [17–20] (Figure 1A). ES cell targeting was verified by genomic Southern blotting (Figure 1B-C adapted from [17]), offspring and embryos were genotyped by PCR (Figure 1D-E). Both *Bapx1*^*tm2.Tlu*^ and *Bapx1*^*tm4.Tlu*^ showed expression of the EGFP protein in the correct *Bapx1*-specific domains (Figure 2A) as previously described [6, 8, 11, 12, 17]. The *Bapx1*^*tm2.Tlu*^ mice were normal when compared to wildtype *Bapx1*^*+/+*^ littermates [20]. Similar constructs were made for the *Sox9* locus to create *Sox9*^*tm1.Tlu*^[20] and *Sox9*^*-/-(EGFP)*^ mice that are described in detail in another manuscript.

### Identification of genes controlled by *Bapx1*in five different tissues

Since *Bapx1* is expressed in multiple tissues in the developing mouse embryo [6, 8, 10, 12, 13], we fine dissected the five principal *Bapx1* domains prior to FACS, namely: vertebral column, spleen, gut, forelimb and hindlimb (Figure 2A). At E12.5 the vertebrate hindlimb is slightly delayed in its development compared to the forelimb [17, 21–24]. To capture the distinct molecular events orchestrated by *Bapx1* in fore- and hindlimbs, we analyzed them separately. Our microarray analysis revealed that *Bapx1* controlled very different sets of genes in all of these five organs, with little overlap between each of the five tissues. While *Bapx1* affected a large number of genes (with >2 fold enrichment) in the spleen (n = 980), gut (n = 143) and vertebral column (n = 125), it controlled only a very limited number of genes in the hind- (n = 30) and forelimb (n = 2) (Figure 2B-C), an observation correlating with the of loss-of function phenotypes in spleen, gut and vertebral column and lack thereof in the limbs, although *Bapx1* is highly expressed during limb development [8–10], and *Bapx1* gain-of-function in the limbs results in polydactyly [13]. Mostly *in vitro* findings indicate primarily a repressive function for *Bapx1*[25–33] and in its absence one would expect increased expression of its primary target genes that it is responsible for silencing.

We then focused on the nature of the regulation by *Bapx1* in each of the three tissues with a loss-of function phenotype (spleen, gut and vertebral column) where *Bapx1* obviously has an important developmental role. In two of these tissues (spleen and gut) *Bapx1* was primarily an activator of downstream genes. We found in *Bapx1-*specific spleen cells 77% of the genes (761/980) and in *Bapx1-*specific gut cells 88% (126/143) of the genes showed decreased expression in the *Bapx1* null embryos. However, in the *Bapx1-*specific vertebral column cells 91% (114/125) of the genes were increased in expression in the *Bapx1* null embryo, indicating that the Bapx1 protein is primarily a repressor of genes during normal vertebral column development (Figure 2B-C & Additional file 1: Table S1) which is consistent with the *in vitro* findings for *Bapx1* mentioned above.

Though it is always possible in multi tissue studies that the varying levels of gene expression changes seen in different organs is a reflection of the different rate of development of each tissue, it is highly unlikely in this case specially in the spleen and limbs. Spleen starts developing as a separate tissue from the surrounding pancreatic mesenchyme at around E12.5 and the role of *Bapx1* in this organ is also important from this time point in development [34]*.* The expression of *Bapx1* in the limb is also first detected at E11.5 [17] and hence we are looking at organs at comparable stages of development. The expression of *Bapx1* in both gut and vertebral column is first detected at E9.5 [11, 17] and comparable numbers of genes are changing in the *Bapx1* null embryo at the stage under investigation. It is also noteworthy that almost 10 times more genes are changing in spleen compared to the gut or vertebral column pointing to the fact that correlation between developmental stage and impact of the loss of transcription factor in an organ can be at times tenuous.

### *Bapx1*represses inappropriate early maturation of chondrocytes

*Bapx1* prevents early maturation of chondrocytes in the vertebral column and its role in vertebral column development is its most studied function. The *Bapx1* null embryos die at birth exhibiting a truncated axial skeleton owing to the absence of ossification centers in the vertebrae, lack of cartilage condensations and a markedly reduced intervertebral disc formation [8–10]. During chondrogenesis, *Bapx1* is considered to primarily suppress the hypertrophic differentiation of chondrocytes [16], but the genes it regulates pertaining to this function are poorly understood. During endochondral ossification, mesenchymal cells of the condensation differentiate into chondrocytes, marked by their ability to secrete Collagen II, IX, XI and Aggrecan, while repressing Collagen type I production. Upon chondrocyte maturation, proliferation of the chondrocytes stops and they progress to hypertrophy, marked by the switch to the production of type X Collagen instead of type II. Terminal hypertrophic chondrocytes then secrete matrix metalloproteinases to degrade the cartilage matrix, facilitating vascular invasion that brings in osteoprogenitors for ossification [35].

**Figure 3:**
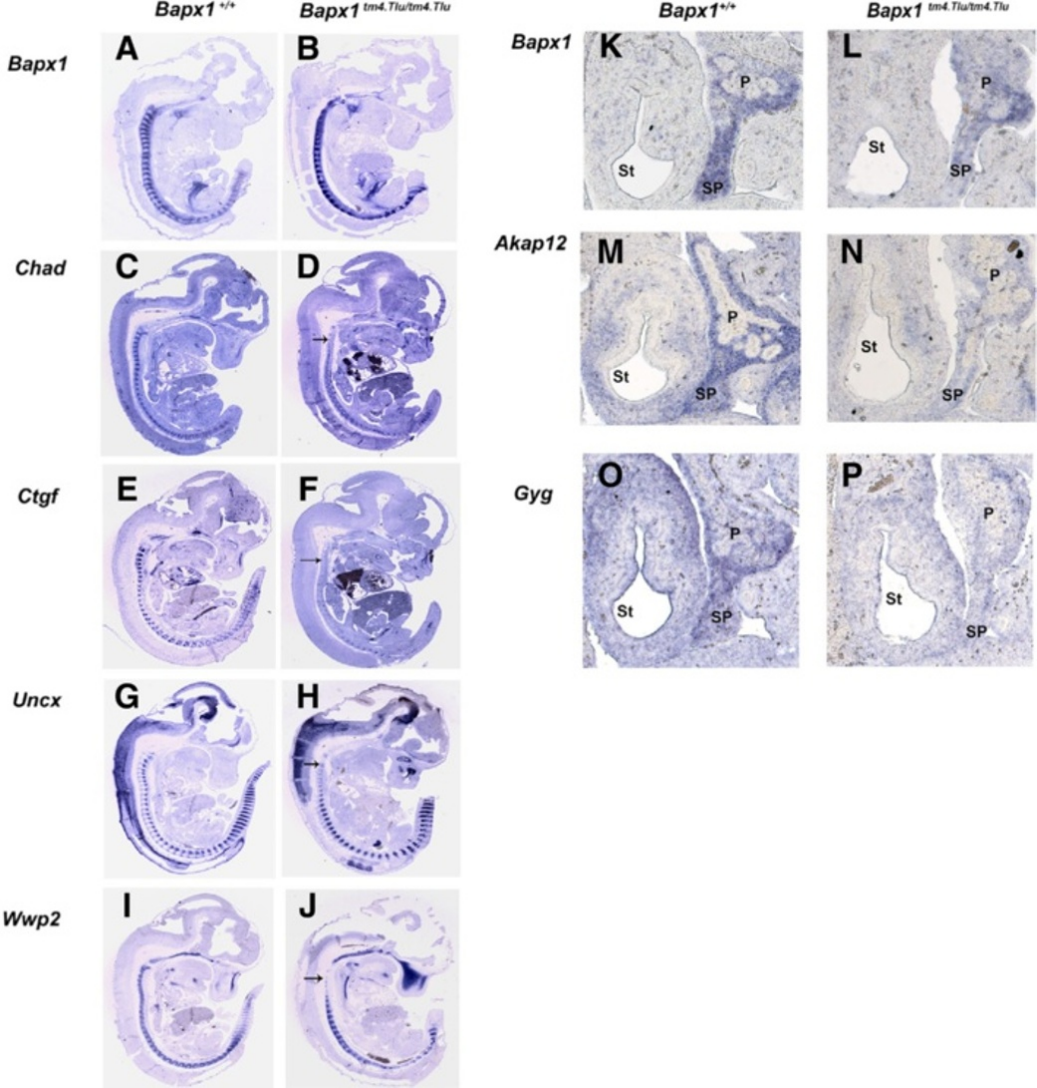
**Validation of Bapx1 target genes by RNA** ***in situ*** **hybridization.** RNA *in situ* hybridization for *Bapx1* regulated genes in E12.5 wildtype and *Bapx1* null embryos validating the expression profiling data for *Bapx1* **(A and B)**, *Chad* **(C and D)**, *Ctfg* **(E and F)** *, Uncx* **(G and H)**, *Wwp2* **(I and J)** in the vertebral column. The arrow marks the loss of expression of the genes in the rostral part of the vertebral column. Similar loss of expression is seen in the spleen of the null embryo for *Bapx1* **(K and L)**, *Akap12* **(M and N)** and *Gyg* **(O and P)**. Abbreviations: Sp, Spleen; St, Stomach; P, Pancreas.

During early axial skeleton development the somite segmentation proceeds from rostral to caudal [35]. Gene ontology (GO) analysis of data from E12.5 embryos using “Database for Annotation, Visualization And Integrated Discovery” (DAVID) [36, 37] revealed enrichment of cartilage condensation/development and neuron development genes. From this, it was evident that *Bapx1* controls key genes in cartilage development like *Uncx*, *Chad, Ctgf* and *Wwp2.* We observed down-regulation of these transcripts in the rostral vertebral column of *Bapx1*-null embryos by *in situ* hybridization (Figure 3A-J). Thus *Bapx1* impacts the level of expression of some chondrogenic genes, but is dispensable for their initiation of transcription. Furthermore, *Bapx1* positively regulates *Sox5*, which regulates extra cellular matrix genes typical for early chondrocyte differentiation like *Col2a1* and *Acan*, in conjunction with *Sox9* and *Sox6*[38, 39]. In this way, *Bapx1* indirectly regulates early chondrocyte differentiation.

At the same time *Bapx1* suppresses neural differentiation by inhibiting genes like *Alcam, Ntrk3, Corin* and *Dcx* and prevents premature osteogenesis by inhibiting osteoblast marker (*Col1a1)*, cartilage degradation genes with metallopeptidase activity (*Ctsk, Adamts4, Adamts2* and *Anpep*) and prevents blood vessel development by inhibiting *Klf5*, *Vegfc* and *Nrp1* (FC ≤ 1.5) (Figure 2D and Additional file 1: Table S1). Correspondingly, Ingenuity Pathway Analysis (IPA) analysis showed enrichment of genes in the Wnt and VEGF signaling pathways, as well as the inhibition of matrix metalloproteases (Additional file 2: Table S2). Wnt/B-catenin signaling is known to suppress chondrogenesis, and cartilage degradation and bone matrix formation are associated with chondrocyte maturation [40]. This points out that *Bapx1*, which largely functions as a repressor in the vertebral column, does not regulate the early differentiation of chondrocytes as much as it represses the inappropriate early maturation of these chondrocytes.

### *Bapx1*controls cell survival in the spleen

*Bapx1* has a strong effect on spleen morphogenesis given the *Bapx1* null embryos are asplenic at birth [8–10]. The underlying mechanism for this phenotype was unclear as genes controlled by *Bapx1* were previously largely unknown. Our transcriptional profiling of *Bapx1* specific spleen cells at E12.5 showed that wildtype *Bapx1* is an activator of a large number of genes involved in cell division and cell cycle like *Bmi1, Cdc14a, Cep55, Ccng2, Lats2, Ccne2, Top1, Stag2, Tfdp1 and Ckap2* (Figure 2E and Additional file 2: Table S2). Moreover, *Nkx2.5,* a known spleen anlage marker and a regulator of spleen growth through the inhibition of cell cycle inhibitor p15, was one of the targets in *Bapx1* null embryos showing loss of expression [41]. *Pdx1*, another spleen development marker is also known to regulate *Nkx2.5. Bapx1* regulates *Nkx2.5* as well, yet without affecting *Pdx1* itself, indicating the possible presence of a parallel pathway that converges at *Nkx2.5* to regulate proliferation of splenic primordial cells [41]. It is further possible that *Pdx1* is upstream of *Bapx1* and mediates *Nkx2.5* via *Bapx1*. Besides *Nkx2.5, Slc40a1* and *Bmi1* were activated by wildtype *Bapx1* and the loss-of function mouse mutants of these genes exhibit a hypoplastic spleen phenotype. Functional ablation of *Slc40a1*[42] results in increased splenocyte apoptosis while *Bmi1* is a cell cycle regulator, which shows impaired splenocyte proliferation upon knock-out [43], suggesting that these genes also mediate the cell viability function of *Bapx1* in the spleen.

IPA analysis predicted the proliferation of cells to be decreased (z-score: -3.922; p: 7.23E-11) while apoptotic genes were enriched (z-score: 1.256; p: 9.23E-06) (Additional file 2: Table S2). *In situ* hybridization showed the presence of remnant splenic primordial mesenchyme at E12.5 in *Bapx1*-null embryos, which is progressively lost with development, resulting in asplenia at birth (Figure 3K-P). Thus, the disruption of cell survival via the deregulation of cell cycle and apoptotic genes in the splenic primordial mesenchyme is likely causative for the hypoplasia of the spleen at E12.5 and its complete absence at later developmental stages in the *Bapx1* null mutants. IPA further revealed the enrichment of genes of the FGF signaling pathway, a pathway essential for cell survival, cell growth, differentiation, morphogenesis and angiogenesis. Other enriched genes included members of the ERK/MAPK signaling, epithelial adherens junction signaling, VEGF signaling and epithelial-mesenchymal transition pathways (Additional file 2: Table S2).

### Validation of novel *Bapx1*target genes during spleen development

We confirmed two genes with no previously attributed role in spleen development by section RNA *in-situ* hybridization (SISH). *Kinase (PRKA) anchor protein (gravin) 12 (Akap12)* previously described in the pancreas [44] was shown here to be expressed and controlled by *Bapx1* in the spleen (Figure 3M-N). Similarly *Glycogenin* (*Gyg)* previously described in the heart and lungs [45], was found to be expressed and controlled by *Bapx1* in the spleen as seen by a significant decrease in expression in the E12.5 day *Bapx1* null spleen (Figure 3O-P). The entire repertoire of genes regulated by *Bapx1* in the spleen would thus serve as a suitable database of splenic development genes for future investigation.

### *Bapx1*controls enzyme inhibitors and viability in the gut cells

*Bapx1* is the mammalian ortholog of the *Drosophila bagpipe*, a gene that is essential for directing the development of the fly visceral mesoderm into gut musculature [46, 47]. In contrast, *Bapx1* does not seem as critical during morphogenesis of the mammalian gut, given most of the previous knock-out studies failed to report a significant gut phenotype, except for a shortening of the antrum-pylorus segment and the loss of the pyloric constriction [8–10]. *Bapx1* expressing cells are reportedly present in the gut mesenchyme with a rostral boundary in the hind stomach, near the junction of the gastric corpus and antrum [11]. By analyzing genes under the control of *Bapx1* we defined its potential role in the gastrointestinal tract. Our GO annotation of genes clearly indicated that *Bapx1* primarily controls genes involved in endopeptidase and other enzyme inhibitor activities (Figure 2E). These include *KngI*, *Ambp*, *Serpina1B, SerpinaA6*, *Spink3* and *Itih3* among others. In analogy to the spleen our IPA analysis revealed that *Bapx1* regulates a significant proportion of proliferation (z-score: -3.054; p: 2.22E-05) and apoptotic genes (p: 3.01E-03) in the gut, thus explaining the shortening of the antrum-pylorus segment in the null embryo (Additional file 2: Table S2). Our genome wide transcriptional profiling revealed genes previously unknown to be controlled by *Bapx1* in the gut and the control of a physiological activity (enzyme inhibitory action) that has not been attributed to it previously. Furthermore, signaling pathways such as ERK/MAPK signaling, VEGF signaling and the epithelial-mesenchymal transition pathway were enriched in the gut similar to our observations in the spleen (Additional file 2: Table S2).

### *Bapx1-Sox9*act synergistically in the developing vertebral column

Previous *in vitro* analyses showed *Bapx1* and *Sox9* connect in an auto-regulatory loop and *Sox9* repressing *Runx2* via *Bapx1*, thereby preventing the inappropriate hypertrophic differentiation of chondrocytes [16]. Hence we investigated the regulatory targets of *Sox9* in the developing E12.5 vertebral column *in vivo*. Similar to our work regarding *Bapx1*, we enriched for *Sox9*-specific cells from the vertebral column of *Sox9*^*+/tm1.1Tlu*^ and *Sox9*^*-/-(EGFP)*^ E12.5 embryos using FACS (Figure 2G). Our microarray data on the *Sox9*-specific cells revealed that the Sox9 protein, unlike Bapx1, is neither an overt activator nor a repressor of transcription in the vertebrate column as 42.2% (1514/3593) genes showed decreased expression (FC1.5; p ≤ 0.05) compared to 57.7% (2073/3593) showing increased expression (FC1.5; p ≤ 0.05) in the *Sox9* null embryo relative to the *Sox9* wildtype embryo (Additional file 1: Table S1). IPA of these *Sox9* controlled genes revealed that most had known roles in chondrocyte differentiation (p: 3.43E-06), proliferation (p: 3.82E-28) and apoptosis (p: 3.92E-14) (Figure 2H and Additional file 2: Table S2) consistent with the known role of *Sox9* as a master regulator of osteo-chondrogenesis. Though many of the downstream genes are known to be involved in chondrogenesis through *in vitro* studies, our data demonstrates that *Sox9* regulates these genes *in vivo* in the developing mouse embryo at E12.5, a time when the developing skeleton is transitioning from mesenchymal condensations to a chondrogenic framework. Similar to the pathways regulated by *Bapx1* in the vertebral column, IPA analysis for *Sox9* regulated genes showed enrichment of the Wnt/B-Catenin and VEGF signaling pathways, and the inhibition of matrix metalloproteases (Additional file 2: Table S2).

**Figure 4:**
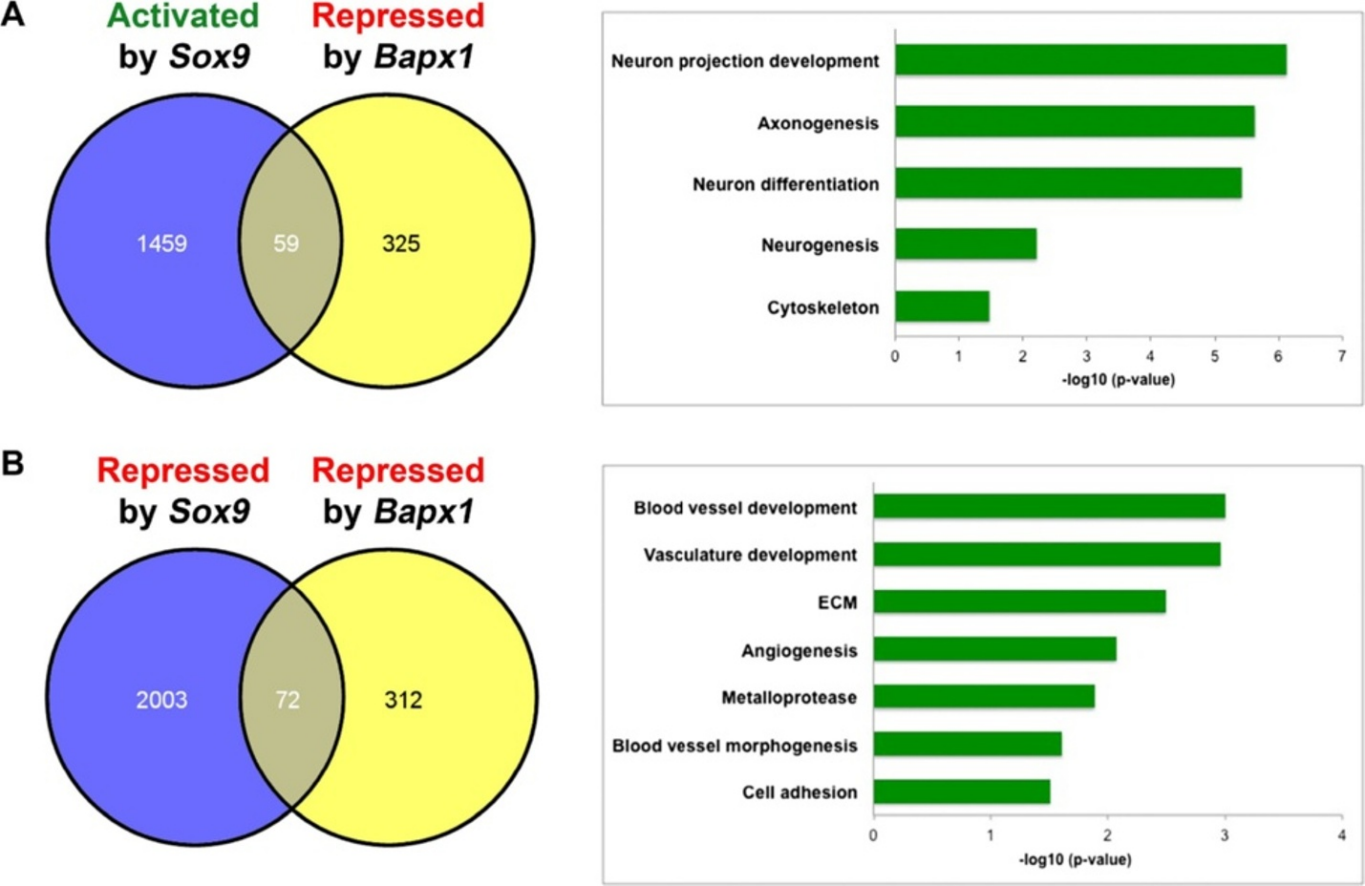
**Co-regulation by** ***Bapx1*** **and** ***Sox9*** **. (A)** Venn diagram showing the 59 common genes activated by *Sox9* and repressed by *Bapx1* in the vertebral column of E12.5 wildtype embryos and the GO annotation of those common genes, which are primarily neuronal markers. **(B)** Venn diagram showing the 72 common genes repressed by both *Sox9* and *Bapx1* in the vertebral column of E12.5 wildtype embryos and the GO annotation of those common genes.

With *Bapx1* and *Sox9* functioning in the same tissue and regulating similar pathways, the question arose whether these two transcription factors act on the same set of genes in a co-regulatory manner. Comparing Sox9 controlled gene expression with Bapx1 target genes allowed us to filter for chondrogenic genes, as *Bapx1* is naturally absent in the neural tube. We focused on three sets of genes: (1) common genes that are activated by *Sox9* but repressed by *Bapx1*, (2) genes repressed by both and (3) genes activated by both. For analysis of gene co-regulated by Sox9 and Bapx1 we have used a cut off of FC1.5 to make a larger gene set to avoid overlooking any gene which might be strongly regulated by one and weakly by other. We found 59 genes to be repressed by *Bapx1* and activated by *Sox9* all primarily involved in neurogenesis and neuronal differentiation like *Gbx2*, *Dbx*, *Nrn1* and *Ctnna2* (Figure 4A and Additional file 3: Table S3).

Analyzing genes repressed by both transcription factors in the vertebral column, we found 72 genes, which included the osteogenic factor *Col1a1*, cartilage degrading metalloproteases (*Mmp11, Adamts2, Adamts4*) and most highly enriched, genes controlling blood vessel development (*Vegfc, Emcn, Nrp1*), an essential process during chondrocyte maturation [48, 49] (Figure 4B and Additional file 3: Table S3 ). Though the role of *Sox9* as a repressor of cartilage vascularization has been shown recently [50], our data suggests that *Bapx1* plays an equally critical role in this process. Thus by repressing these genes at an early time point in development it prevents premature ossification of the developing vertebral column. Moreover, *Bapx1* and *Sox9* function as activators on only six common target genes, including *Sox5, Wwp2, Chad* and *Ctgf*, which are all known to be involved in cartilage development [14, 50–52] (Additional file 3: Table S3).

Surprisingly, we did not detect *Bapx1* as one of the downstream targets of *Sox9* or vice versa at E12.5 in the vertebral column (Additional file 1: Table S1 and Additional file 3: Table S3). However, we found *Sox9* to be downstream of *Bapx1* in the spleen and gut tissue. Besides, *Bapx1* was clearly upregulated in the *Bapx1-null* embryos in the vertebral column (Figure 3A, B), indicating potential auto-regulation.

### Genome wide binding of Bapx1 and Sox9 proteins to DNA target sites in the vertebral column

**Figure 5:**
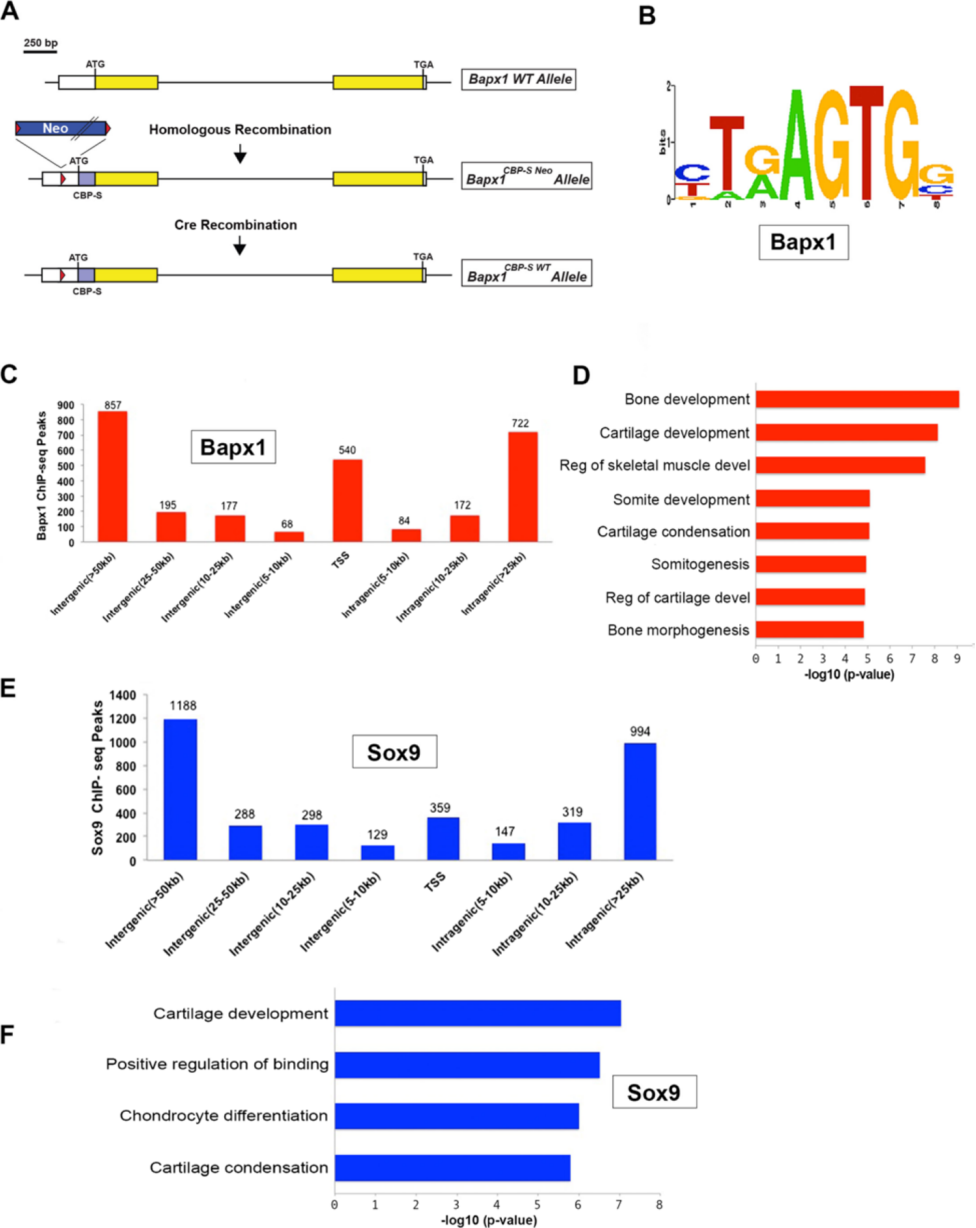
***In vivo*** **ChIP-Seq for Bapx1 and Sox9 in the vertebral column of E12.5 embryos. (A)** Targeting of the endogenous *Bapx1* locus with a CBP- S peptide tag to generate N-terminal tagged BAPX1 protein. **(B)** The Bapx1 binding site motif enriched in the top 1000 binding sites from ChIP-Seq. **(C)** Whole genome binding site distribution for Bapx1 in the vertebral column of E12.5 wildtype embryos. The number on top of each peak represents the total number of peaks found at that distance from the TSS. Note that most peaks are found distally from the TSS. **(D)** GO annotation of the nearest genes to the peak shows enrichment of genes expressed in chondrocytes. **(E)** Similar binding site distribution for Sox9 and **(F)** GO annotation for the nearest genes showing enrichment of genes expressed in chondrocytes, x-axis is -log10 (p-value).

The pool of differentially expressed genes from the expression profiling consisted of target genes that were regulated directly by Bapx1 and Sox9, and indirect target genes whose expression was altered owing to the loss of these two transcription factors affecting their upstream regulators (secondary effectors). To find genes directly controlled by Bapx1 and Sox9 proteins during vertebral chondrogenesis, we performed chromatin immunoprecipitation sequencing (ChIP-Seq) on vertebral columns dissected from E12.5 embryos. For Sox9, we performed ChIP-Seq with an anti-Sox9 antibody. However for Bapx1, owing to the absence of a suitable ChIP grade quality Bapx1 antibody, a mouse line with a S-Peptide and Calmodulin binding peptide (CBP) epitope tagged to Bapx1 at its N-terminus was generated (Figure 5A and Additional file 4: Figure S1) similar to other work we have described [53–56]. S-peptide is a 15 amino acid tag and CBP is a 26 amino acid tag, routinely used for protein purification purposes [57–59] and owing to their small size suitable for endogenous tagging of transcription factors in the mouse [53, 54]. Their absence from the wildtype mouse proteome allows for highly specific ChIP. The homozygous Bapx1 protein tagged mice we generated were phenotypically normal compared to their wildtype littermates, assuring normal biological activity of the tagged protein and thus allowed us to use the S-peptide antibody to pull down tagged Bapx1.

Using the model-based analysis for ChIP-Seq (MACS) algorithm [60] we detected 2815 Bapx1 peaks with 19% (540) of the binding sites located on the transcription start sites (TSS) of genes (Figure 5C). Interestingly a large proportion of the binding sites were distal from the TSS, with 37% (1052) located at >25 kb distally in the intergenic region and 26% (722) located >25 kb in the intragenic regions (Figure 5C). Likewise for Sox9, we identified 3722 peaks. Similar to Bapx1, most of the binding sites were located distal to the TSS, with only ~ 10% (359) of the Sox9 binding sites at the TSS of various genes and 66% (2470) of the binding sites more than 25 kb distal to the TSS (Figure 5E). This shows that in midgestation embryos, critical transcription factors like Bapx1 and Sox9 tend to bind primarily to distal regulatory elements in order to direct downstream gene expression in target cells and tissues rather than activating transcription at the TSS. To ascertain which are potential Bapx1 direct targets in the vertebral column we looked at genes that are differentially expressed (fold change = 1.5) in the microarray dataset for the vertebral column and discovered that of all the genes that have a binding site within 100 kb of their TSS 84% are up-regulated in the *Bapx1-null*. This adds confidence to the data that the Bapx1 protein primarily acts as repressor in the vertebral column (Additional file 5: Table S4).

We identified the previously reported Bapx1 binding motif T(G/A)AGTG [28], enriched in about 40% of the Bapx1 binding sites in the genome (Figure 5B) specifically near genes controlling cartilage and bone morphogenesis (Figure 5D). Notably, this motif was reported to be involved in chondrogenesis *in vitro*, in cell culture and chicken embryo explant assays [28, 29, 61]. Our data is the first to demonstrate the *in vivo* binding of Bapx1 to genomic DNA by this motif in mouse embryo chondrogenic cells. We further validated the Bapx1 binding motif by performing electrophoretic mobility shift assays (EMSA) with four randomly selected genomic regions containing this motif (Additional file 6: Table S5). This allowed us to test the binding specificity of the motif in its native context. All four of the randomly chosen genomic regions containing the motif were able to bind Bapx1 *in vitro* (Additional file 7: Figure S2A). To further confirm the specificity of the motif, we mutated all the nucleotides of the core sequence of one of the regions by transversion and noted a complete abrogation of binding (Additional file 7: Figure S2B). We ascertained the specificity of this binding of the probe to Bapx1 using a supershift assay. We generated NIH3T3 cells overexpressing V5-tagged Bapx1 and used an anti-V5 antibody on nuclear extract for specificity and an anti-Flag antibody as a non-specific control. Our results showed a supershift with the anti-V5 antibody, whereas the addition of anti-Flag antibody had no effect on the shift (Additional file 7: Figure S2B). We further analyzed if some of the Bapx1 bound regions could act as enhancers in a transactivation assay using a luciferase reporter. We cloned seven randomly selected genomic regions (including the four tested in EMSA) (Additional file 6: Table S5) in a minimal promoter driven luciferase vector (pGL4.23) and co-transfected it with the V5-tagged Bapx1 vector into NIH3T3 cell lines. Five out of the seven regions showed activity over the basal level thus acting as an enhancer. To further test if the core-binding motif for *Bapx1* was necessary and sufficient for this activity we used mutated oligonucleotides for the core sequence in two of the regions. The mutated oligonucleotides failed to drive expression over the basal level, thus proving that the core Bapx1 motif is the essential component of these enhancers (Additional file 7: Figure S2C). Similarly, most of the genes nearest to the Sox9 binding regions were involved in chondrogenesis, validating the specificity of the data (Figure 5F). We also identified the DNA binding motif for Sox9 (A/T)(A/T)CAA(A/T)G that had been previously identified mostly by *in vitro* studies [62–64]. Our data shows that Sox9 binds near genes involved in chondrogenesis in the developing mouse embryo by this same motif.

### Direct co-regulation by Bapx1 and Sox9 in the vertebral column

Next we established how many of the genes in these groups have binding sites for both transcription factors within 200 kb of their TSS. Of the group of genes activated by Sox9 and repressed by Bapx1, 86% (51/59) had both the Bapx1 and Sox9 binding sites within 200 kb of their TSS (Additional file 8: Table S6). These genes represent potential primary targets of Bapx1 and Sox9, which are under opposite regulatory pressure in the vertebral column to maintain a distinct neuronal and osteo-chondrogenic identity in the developing embryo, they include genes like *Gata3, Ctnna2, Nrn1* and *Dcx*, whose knockouts have a reported neuronal phenotype [65–69] but have never been associated with a regulation by either Sox9 or Bapx1. Our dataset thus connects Bapx1 and Sox9 downstream genes with a dual regulatory mechanism. Similarly, 78% (56/72) of the genes repressed by both Sox9 and Bapx1 had binding sites for both Sox9 and Bapx1 within 200 kb of their TSS (Additional file 3: Table S3 and Additional file 8: Table S6). These genes are also potential primary targets of both Sox9 and Bapx1, but repressed by both. This group includes the osteogenic, metalloproteinase and angiogenesis genes *Col1a1, Mmp11, Adamts4, Ctsk, Emcn* and *Nrp1*. We looked for binding sites that were overlapping for both the transcription factors but failed to find any at this stage of development. This was not entirely unexpected since both are large transcription factors with distinct motifs thus it is slightly unlikely they could occupy a single enhancer without causing steric hindrance at the same time in development.

**Figure 6:**
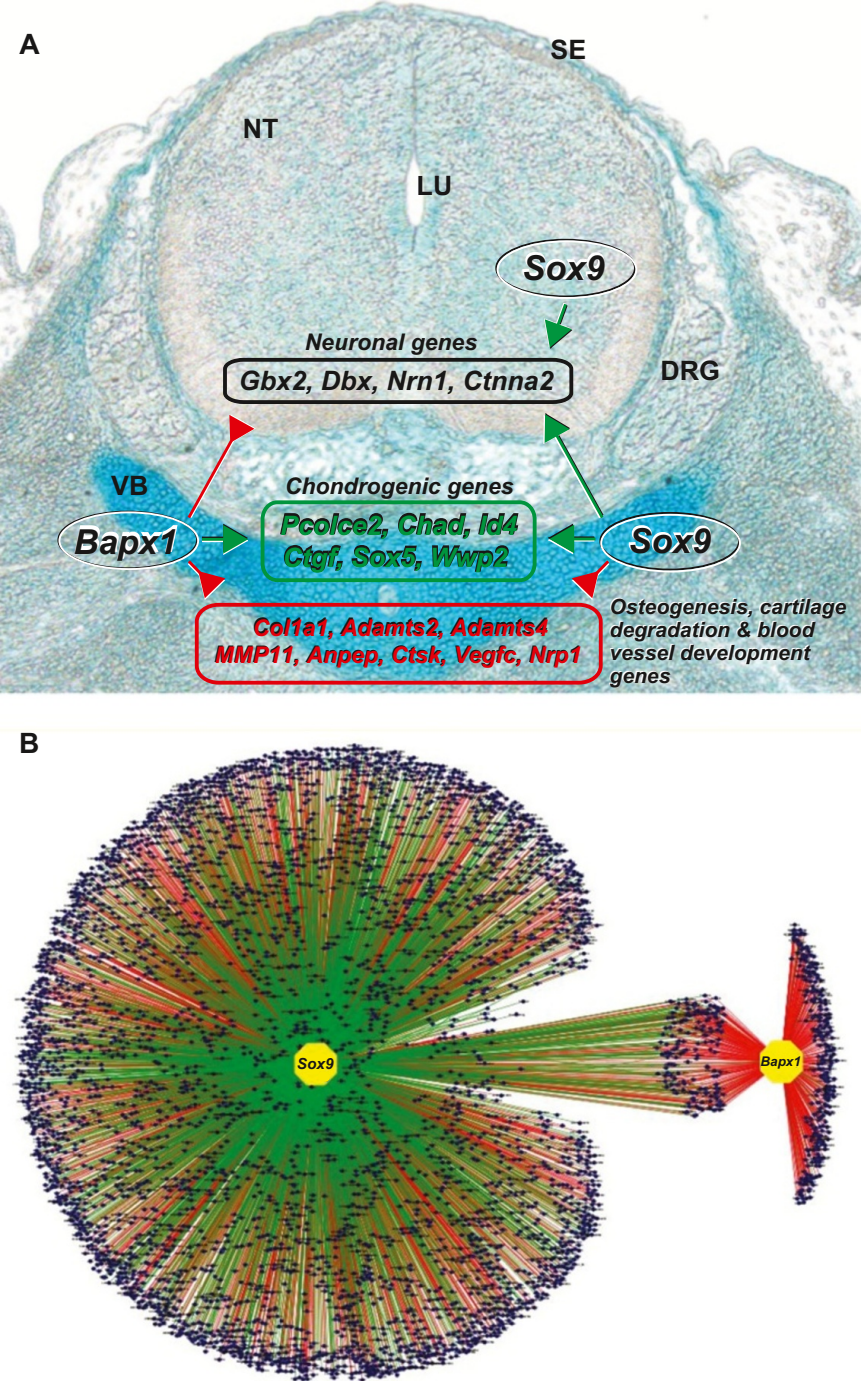
**A gene regulatory network in vertebral column driven by** ***Bapx1*** **and** ***Sox9*** **. (A)** Three layer mechanism of genomic control in the vertebral column by *Bapx1* and *Sox9. Bapx1* suppresses neuronal genes whereas *Sox9* activates them; both activate chondrogenic genes whereas both suppress genes involved in osteogenesis, cartilage degradation and blood vessel development. **(B)** A large gene regulatory network in the vertebral column of genes that are either activated (green lines) or repressed (red lines) by *Sox9* and *Bapx1*. Each blue dot represents a gene and the common regulated genes are in the center on the network connected to both *Sox9* and *Bapx1*.

Our results show that combining genome wide data for binding and transcriptional profiling of two critical transcription factors (Bapx1 and Sox9) in a single organ can lead to a detailed understanding of the diverse levels of regulation involved in its organogenesis. Some neuronal genes were found under a competing control mechanism, receiving both positive and negative inputs: primarily neuronal genes and genes controlling processes essential for chondrocyte maturation were repressed in chondrocytes and those promoting chondrogenesis were activated by both Bapx1 and Sox9 (Figure 6A). Finally by assembling information from all the genes regulated by Bapx1 and Sox9 either directly or indirectly a large-scale integrative gene regulatory network could be delineated (Figure 6B).

## Discussion

It is becoming increasingly clear that an in-depth understanding of organogenesis in vertebrates will require comprehensive systems biology approaches to tease out all the players in various pathways and integrate them into a mechanistic network. Gene expression profiling coupled with genome wide binding studies for particular organs have over the years shed some light on these complex processes [70, 71]. However, transcriptome analysis of an entire tissue or organ only gives a broad and often confusing picture of the precise transcriptional changes taking place during development owing to a large heterogeneity of different cell types that comprise individual tissues that make up a functional organ. By conducting expression profiling only on cells that express a particular set of transcription factors we generated a highly focused dataset elucidating pleiotropic roles played by the transcription factors in organogenesis.

We have analyzed five different organs expressing the *Bapx1* gene to explain the disparate roles it plays in each of them. Although the pleiotropic role of *Bapx1* was known and many phenotypes defined, our approach is the first large-scale effort looking at all the major tissues expressing *Bapx1*. This allowed us to make connections between many new genes previously unknown to be controlled by *Bapx1*. The large scale dataset generated enabled us to place these genes within pathways they control, essentially explaining the lack of a limb phenotype, the absence of an overt morphological phenotype in the gut and the impact on cell cycle regulation in the spleen leading to asplenia in the *Bapx1* loss-of-function mice. Importantly, *Bapx1* largely regulates different genes and pathways in the different tissues to execute its pleiotropic roles. Interestingly, spleen and gut tissue, which are closely associated during gastrointestinal development [72] share more similar pathways with each other than with vertebral column tissue. Indeed, among the five tissues, spleen and gut share the most number of common genes. Intriguingly, while Bapx1 represses VEGF signaling in the vertebral column and gut, it is largely activated in the spleen. The spleen is a repository of red blood cells and its development involves vascularization that allows the infiltration of lymphoid progenitors as early as E12.5-E13.5 [73]. Hence, positive regulation of angiogenesis in the spleen is not too surprising. At the same time, consistent with its role in preventing premature endochondral ossification, Bapx1 inhibits VEGF signaling in the vertebral column.

The formation of gene regulatory networks in vertebrate development continues to be a long arching goal of developmental biology. Yet, to establish such detailed maps, many times input from more than one transcription factor is required. Hence, we co-analyzed in similar fashion cells specific for *Sox9*, another key transcription factor during chondrogenesis and vertebral column development. Combining the genes regulated by Bapx1 and Sox9 in the vertebral column we have generated a network of genes activated and/or repressed by these transcription factors during the fine tuned process of organogenesis. We have identified 137 genes co-regulated by these two transcription factors providing insight into some of the mechanisms employed during chondrocyte maturation; there is a group of neuronal genes, which are acted upon antagonistically by *Bapx1* and *Sox9,* chondrogenic differentiation genes which are activated by both *Sox9* and *Bapx1,* and a group of genes which are repressed by both *Sox9* and *Bapx1* that are involved in processes essential for hypertrophic chondrocyte differentiation. Furthermore, *Bapx1* and *Sox9* contribute to several similar pathways during vertebral column development, especially the repression of osteogenic differentiation and cartilage degradation. We also studied the genome wide binding of Bapx1 and Sox9 in vertebral column tissue to identify potential primary target genes. Our ChIP-Seq data revealed that although Bapx1 and Sox9 bound to the TSS of some genes, the majority of the genes regulated by these two transcription factors had their binding sites distal to the TSS, highlighting the fact that during the midgestation development in mouse embryos many critical transcription factors bind to distal *cis*-regulatory elements.

## Conclusions

In all we have managed to create a comprehensive list of genes controlled by a single transcription factor namely Bapx1 from five different tissues, explaining its diverse roles and the various genetic pathways Bapx1 contributes to. This is the first study analyzing all major Bapx1 dependent organs in molecular detail and providing explanations for the phenotypes observed in the loss-of-function animals. Many of the here identified target genes were previously unknown to be either directly or indirectly regulated by *Bapx1* owing to a lack of feasible strategies for determining downstream effectors on a genome wide scale. This genomic resource and the underlying mechanisms uncovered will form the basis for a deeper understanding of the complex process of organogenesis in the developing mammalian embryo.

## Methods

### Ethical statement

All animal procedures were performed according to the Singapore A*STAR Biopolis Biological Resource Center (BRC) Institutional Animal Care and Use Committee (IACUC) guidelines which are set by the National Advisory Committee for Laboratory Animal Research (NACLAR). The IACUC protocols employed were reviewed and approved by the aforementioned committee before any animal procedures were undertaken for this study described here (IACUC Protocol No: 110689 and 110648). The mouse strains used in this study were housed, maintained and provided by the A*STAR Biopolis Biological Resource Center. The lines described here will be made available to the research community upon acceptance of the manuscript.

### Gene targeting and mouse generation

The over 160 kb murine BAC clone RP24-148P5 containing genomic C57BL/6 J DNA flanking the *Bapx1* gene was obtained from the BACPAC Resources Centre at Children’s Hospital Oakland Research Institute (CHORI) and modified using a Quick and Easy BAC modification kit (Gene Bridges) according to the manufacturer’s instruction. The *Bapx1*^*tm2.Tlu*^ allele was generated as described in [17] to generate wildtype mice expressing EGFP in the *Bapx1* domains. The *Bapx1*^*tm4.Tlu*^ allele was generated by inserting the EGFP-FRT-PGK-gb2-Neo-FRT cassette immediately after the translational start codon of *Bapx1*, deleting 90 bp of the endogenous sequence to create the *Bapx1* null mice expressing EGFP. The gene targeting events were confirmed by Southern blotting and for each allele, at least two independent ES cell clones with normal karyotype were subsequently microinjected into 2-8-cell stage embryos isolated from C57BL/6 mice to generate chimeric mice as previously described [74]. The chimeric mice were bred to wildtype mice to generate stable lines. The *FRT*-flanking neomycin cassette in the targeted *Bapx1* allele was removed by breeding to the *FLPe*–deleter mice 129S4/SvJaeSor-*Gt(ROSA)26Sor tm1(FLP1)Dym /*J (Stock # 3946) from Jackson Laboratories [75, 76]. Routine PCR genotyping of *Bapx1* lines was performed as essentially described in [17].

### Sorting Bapx1-EGFP cells from mouse embryos

The mouse embryos were harvested in ice-cold Leibovitz L-15 medium (Gibco) at E12.5. Embryos expressing EGFP in the *Bapx1*-expression domains were identified under a fluorescent dissection microscope (Leica). The embryos were dissected to separate out the vertebral column, spleen, gut, hindlimb and forelimb, which were then separately dissociated into single cells with a solution comprising of 100U/ml Collagenase I & II, 50U/ml DNAse and 0.05% Trypsin (Invitrogen). The cells were filtered serially through a 100uM and 40uM cell strainer, and centrifuged at 2000 rpm for 5 min. The cell pellet was resuspended in 5% FBS, 4 mM EDTA in Leibovitz L-15 medium for cell sorting using FACSAria (BD Biosciences).

### Gene expression microarrays and analyses

Total RNA was extracted form 4 biological replicates for both wild type and null embryos for each of the tissues. Each biological replicate was isolated from each population of sorted cells using TRIzol (Invitrogen) followed by the RNeasy Micro Kit (Qiagen). Sample integrity was assessed using an Agilent 2100 Bioanalyzer (Agilent Technologies). 25 ng total RNA from each sample was labelled using TargetAmp™-Nano Labelling Kit for Illumina Expression BeadChip (Epicentre Biotechnologies) and hybridized on MouseWG-6 v2.0 Expression BeadChip microarrays (Illumina) according to Illumina guidelines. GenomeStudio software (Illumina) was used to prepare background-subtracted data. The background-subtracted data was then imported into Partek Genomics Suite (Agilent) and the data normalized. The probe intensities from various biological replicates were normalized using scale normalization using median absolute deviation as a spread measure. This was further filtered on percentile (lower 20 and upper 100) by expression of probe sets without averaging over replicates. These consistent probe sets, which passed this filter, were further used to calculate gene expression changes between different conditions. ANOVA with nominal alpha value set to 0.05 was then used to determine the probe sets significantly different between the different genotypes compared. Benjamini and Hochberg Multiple testing correction was applied to reduce the false positive rate, and probe sets with expression fold change >2.0 or >1.5 between the different genotypes were selected for further validation and analyses. All data has been deposited in Gene Expression Omnibus (GEO) under accession number GSE35877. Ingenuity Pathway Analysis (Ingenuity® Systems, http://www.ingenuity.com) and DAVID (http://david.abcc.ncifcrf.gov/) gene ontology analyses were performed using the respective web-based tools.

### ChIP-assay and peak calling

For Bapx1 ChIP vertebral columns from ~100 E12.5 (both S-peptide tagged and wildtype each) embryos were dissected and 2 mg of chromatin was used for ChIP as previously described [77] with anti S-Peptide antibody (Bethyl laboratories, A190-134A). For Sox9 ChIP anti-Sox9 antibody (R&D Systems, AF3075) was used for immunoprecipitation. 10-15 ng of purified ChIP DNA from each sample was used to synthesize the sequencing library as instructed by the ChIP-Seq DNA sample Prep Kit (Illumina). The libraries were then subjected to Solexa sequencing according to Illumina’s instruction. Sequence reads produced by Illumina Genome Analyzer II/IIx that passed the signal purity filtering were mapped to the mouse genome mm9, using the Illumina Genome Analyzer Pipeline. All uniquely mapped reads that are with two or fewer mismatches were retained. Genomic binding sites in the ChIP-Seq datasets were identified using the peak calling algorithm MACS (version 1.4.0 beta) with default settings (band width = 300, model fold = 10, 30, p-value cutoff = 1.00e-05, range for calculating regional lambda = 1000 and 10000 bps) [62]. The corresponding control libraries were used for all the peak callings. For Annotation of the ChIP-Seq peaks, first the TSS of the nearest Refseq gene nearest to peaks were found, then the distance from nearest TSS was found and reported. If the peaks resided in intragenic region of nearest gene it was called “intragenic”, otherwise “intergenic”. If the peaks resided within 5000 bp of TSS they were called as “promoter” peaks. All data has been deposited in Gene Expression Omnibus (GEO) under accession number GSE35877 and also provided in BED format (Additional file 9: Table S7).

### Motif analysis

Peaks called by MACS were ranked according to the total tags count as defined in the MACS output file. The top 200 peaks were used for motif analysis and the repeat masked genome sequence +/- 50 bp from the summit of these 200 peaks was downloaded from the UCSC genome browser (http://genome.ucsc.edu/). After masking repeats to N, we performed *de novo* motif finding using MEME ver. 4.3.0 with the sequences. MAST was used to scan for the occurrences of the primary *de novo* motif obtained using all the sequences +/-50 bp of the ChIP-Seq peak summit. The cut off for motif match in MAST used was the default p = 1.0e-4.

### Gene enrichment test for regions bound by Bapx1 and Sox9

We submitted the analyzed ChIP-Seq data as bed format to Genomic Regions Enrichment of Annotations Tool (GREAT) [78] version 1.8 with the following parameters- Species assembly-mm9 and Association rule-Single nearest gene: 100,000 bp maximum extension, curated regulatory domains included.

### RNA *in-situ*hybridization

*In situ* hybridizations were performed using DIG labeled RNA probes as previously described [17]. For analysis of the vertebral column the E12.5 embryos were sectioned through the sagittal plane and for analysis of the spleen through the dorsofrontal plane.

### Luciferase reporter assay and EMSA

Luciferase assay was performed to determine the transcriptional activation property of some of the binding sites bound by Bapx1. Mouse *Bapx1* full-length cDNA (40131175, Openbiosystems) was cloned upstream of the V5-tag in the pcDNA6/V5-HisABC vector (Invitrogen). 400 ng of firefly luciferase vector (pGL4.23, Promega Corporation) containing the binding sites, 500 ng each of *Bapx1* expression vector, and 2 ng of Renilla luciferase vector (transfection control) was transiently transfected into mouse fibroblast cell line (NIH/3 T3) (5 – 6 × 10^4^ cells/well) using 6ul of FuGENE HD transfection reagent (Roche Diagnostic, USA), in 100 μl of OPTI-MEM I medium (Invitrogen, USA). The cells were grown for 48 hours and the luminescence was measured using Dual Luciferase Reporter Assay System on a Glomax multidetection system Luminometer as per manufacturer’s instructions (Promega Corporation).

Electrophoretic mobility shift assays were performed as previously described [79]. Briefly V5 tagged Bapx1 vector was transfected in NIH/3 T3 cells using FuGENE HD transfection reagent. Cells were harvested 48 hours post transfection and nuclear proteins were extracted using the NE-PER nuclear and cytoplasmic extraction kit (Thermo Scientific, USA). EMSAs were carried out using 10 nM DNA probes modified with 5’ Cy5 labels (Sigma Proligo) incubated with 10ug nuclear extracts. For supershift assays the probe and the nuclear extract were incubated with 1ug of V5 antibody (ab15828, Abcam) or Flag (F7425, Sigma) was used as a non-specific antibody.

### Gene regulatory network

The gene regulatory network was constructed using Cytoscape [80], by considering all genes that are showing >1.5 fold change in the microarray (activated as well as repressed).

## Electronic supplementary material

Additional file 1: Table S1: *In vivo* gene expression changes. Obtained from microarrays on gene specific cell lineages obtained from wildtype and loss of function vertebrate columns at E12.5. Gene expression changes in all five tissues with >2 fold difference for *Bapx1* and >1.5 fold difference for *Sox9* and *Bapx1*. (XLSX 189 KB)

Additional file 2: Table S2: Ingenuity Pathway Analysis. Ingenuity Pathway Analysis of *Bapx1* in the spleen, vertebral column and gut and *Sox9* in the vertebral column. (XLSX 17 KB)

Additional file 3: Table S3: Genes co-regulated by *Sox9* and *Bapx1* in vertebral column. Common genes either repressed by both *Sox9* and *Bapx1*, activated by *Sox9* and repressed by *Bapx1* or activated by both in the E12.5 vertebral column >1.5 fold. (XLSX 45 KB)

Additional file 4: Figure S1: Targeting of the murine *Bapx1 locus.* N-terminal targeting of the murine *Bapx1* locus with an S-peptide/CBP tag. (PDF 106 KB)

Additional file 5: Table S4: Genes with Bapx1 binding site within 100 kb of the TSS. (XLSX 11 KB)

Additional file 6: Table S5: Probe and DNA binding sequence for the EMSA and luciferase assays. (XLSX 9 KB)

Additional file 7: Figure S2: EMSA and Luciferase assay Bapx1 bound genomic regions. (A) EMSA gel showing shifting of four different probes (black arrow) containing genomic loci with the Bapx1 binding motif in presence of nuclear extract containing over-expressed V5-tagged Bapx1. FP, free probe. (B) Gel showing mutant probe (MT) with no shift, and incubation with V5 antibody (V5Ab) resulting in a supershift (black arrow) and no supershift with a non-specific Flag antibody (FlagAb). The white arrow indicates the shift. (C) Luciferase assay with seven genomic regions containing the Bapx1 binding motif (R1-R7) and two mutant probes (M1-M2) for regions R3 and R7. (PDF 240 KB)

Additional file 8: Table S6: Binding site distance from the TSS. Binding site distance from the TSS for *Bapx1* and *Sox9* near genes co-regulated by both in vertebral column. (XLSX 21 KB)

Additional file 9: Table S7: ChIP coordinates in BED format. (XLSX 452 KB)

## Abbreviations

CBP: Calmodulin binding peptide
ChIP-Seq: Chromatin immunoprecipitation sequencing
EGFP: Enhanced green fluorescent protein
EMSA: Electrophoretic mobility shift assay
ES: Embryonic stem (cell)
FACS: Fluorescence activated cell sorting
GO: Gene ontology
GRN: Gene regulatory network
IPA: Ingenuity Pathway Analysis, TSS, Transcriptional start site
TSS: Transcriptional start site.
