# Supplementary figures and images for: In vivo genome-wide analysis of multiple tissues identifies gene regulatory networks, novel functions and downstream regulatory genes for Bapx1 and its co-regulation with Sox9 in the mammalian vertebral column
Source: BMC Genomics. 2014 Dec 5;15(1):1072. doi: 10.1186/1471-2164-15-1072 (PMC4302147; doi:10.1186/1471-2164-15-1072)

Supplementary Figure 1

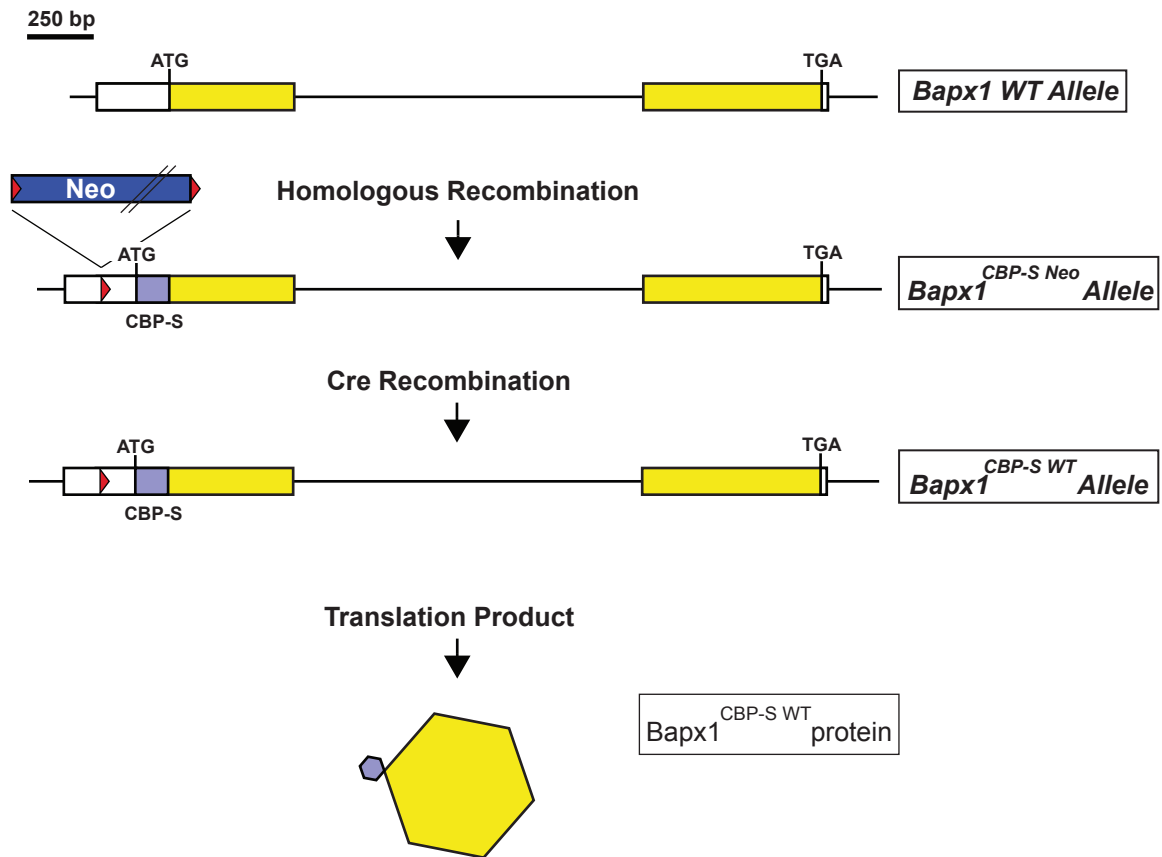

Supplement: Supplementary file 4 — Additional file 4: Figure S1: Targeting of the murine Bapx1 locus. N-terminal targeting of the murine Bapx1 locus with an S-peptide/CBP tag. (PDF 106 KB) [file 12864_2014_6852_MOESM4_ESM.pdf]

**A**

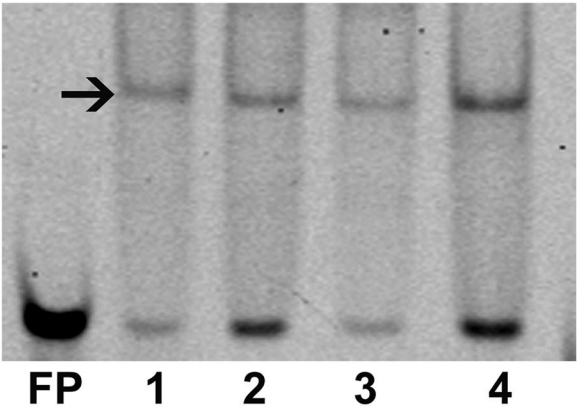

**B**

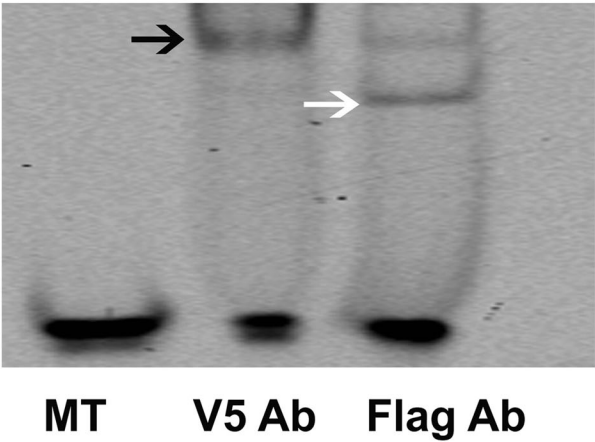

**C**

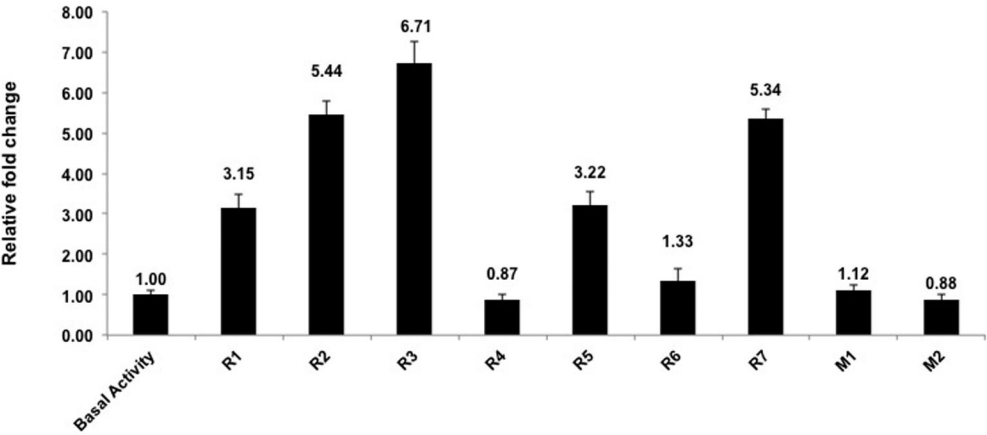

Supplement: Supplementary file 7 — Additional file 7: Figure S2: EMSA and Luciferase assay Bapx1 bound genomic regions. (A) EMSA gel showing shifting of four different probes (black arrow) containing genomic loci with the Bapx1 binding motif in presence of nuclear extract containing over-expressed V5-tagged Bapx1. FP, free probe. (B) Gel showing mutant probe (MT) with no shift, and incubation with V5 antibody (V5Ab) resulting in a supershift (black arrow) and no supershift with a non-specific Flag antibody (FlagAb). The white arrow indicates the shift. (C) Luciferase assay with seven genomic regions containing the Bapx1 binding motif (R1-R7) and two mutant probes (M1-M2) for regions R3 and R7. (PDF 240 KB) [file 12864_2014_6852_MOESM7_ESM.pdf]
